# Impact of MSMEG5257 Deletion on *Mycolicibacterium smegmatis* Growth

**DOI:** 10.3390/microorganisms12040770

**Published:** 2024-04-11

**Authors:** Ping He, Bing Zhao, Wencong He, Zexuan Song, Shaojun Pei, Dongxin Liu, Hui Xia, Shengfen Wang, Xichao Ou, Yang Zheng, Yang Zhou, Yuanyuan Song, Yiting Wang, Xiaolong Cao, Ruida Xing, Yanlin Zhao

**Affiliations:** 1 Chinese Center for Disease Control and Prevention, Changping District, Beijing 102206, China; heping201511401200@yeah.net (P.H.); zhaobing@chinacdc.cn (B.Z.); hewc2312@163.com (W.H.); szx616101@163.com (Z.S.); liucommon@126.com (D.L.); xiahui@chinacdc.cn (H.X.); wangsf@chinacdc.cn (S.W.); ouxc@chinacdc.cn (X.O.); zhengyang@chinacdc.cn (Y.Z.); zhouyang@chinacdc.cn (Y.Z.); songyy@chinacdc.cn (Y.S.); wangyitinglz@163.com (Y.W.); caoxiaolong521jing@outlook.com (X.C.); xingrd@chinacdc.cn (R.X.); 2 School of Public Health, Peking University, Haidian District, Beijing 100871, China; psj22@bjmu.edu.cn

**Keywords:** *Mycolicibacterium smegmatis*, *msmeg5257*, ABC transporter, membrane protein, bacterial metabolic

## Abstract

Mycobacterial membrane proteins play a pivotal role in the bacterial invasion of host cells; however, the precise mechanisms underlying certain membrane proteins remain elusive. *Mycolicibacterium smegmatis* (Ms) *msmeg5257* is a hemolysin III family protein that is homologous to *Mycobacterium tuberculosis* (Mtb) *Rv1085c*, but it has an unclear function in growth. To address this issue, we utilized the CRISPR/Cas9 gene editor to construct Δ*msmeg5257* strains and combined RNA transcription and LC-MS/MS protein profiling to determine the functional role of *msmeg5257* in Ms growth. The correlative analysis showed that the deletion of *msmeg5257* inhibits ABC transporters in the cytomembrane and inhibits the biosynthesis of amino acids in the cell wall. Corresponding to these results, we confirmed that MSMEG5257 localizes in the cytomembrane via subcellular fractionation and also plays a role in facilitating the transport of iron ions in environments with low iron levels. Our data provide insights that *msmeg5257* plays a role in maintaining Ms metabolic homeostasis, and the deletion of *msmeg5257* significantly impacts the growth rate of Ms. Furthermore, *msmeg5257*, a promising drug target, offers a direction for the development of novel therapeutic strategies against mycobacterial diseases.

## 1. Introduction

Tuberculosis (TB) is a disease caused by *Mycobacterium tuberculosis* (Mtb). The global tuberculosis mortality in 2022 reached a staggering figure of 1.3 million deaths [1]. The escalating prevalence of multidrug-resistant TB (MDR-TB) has presented an amplified challenge in managing tuberculosis within clinical settings [2,3]. Meanwhile, in the context of increasingly complex TB care, encompassing the escalating prevalence of extensively drug-resistant TB (XDR-TB) and co-infection with other ailments, such as human immunodeficiency virus (HIV) or malaria [4], the successful treatment of TB is further compounded [5]. The outer membranes of mycobacteria exhibit low permeability, which confers antibiotic resistance upon them [6].

Mycobacteria possess a remarkably complex cell envelope consisting of a cytoplasmic membrane and a cell wall, constituting an efficient permeability barrier, which plays a crucial role in intrinsic drug resistance and survival under harsh conditions [7]. *Mycobacterium smegmatis* (Ms) MmpL3 mutant strains exhibit alterations in cell wall hydrophobicity and growth defects, resulting in resistance to two anti-tubercular agents, SQ109 and AU1235 [8]. The previous study reported that the Rv2686c-Rv2687c-Rv2688c operon, which encodes an ABC transporter in Mtb, conferred Ms resistance to ciprofloxacin [9]. The complexity and abundance of glycoconjugates represent the most distinguishing characteristics of the mycobacteria cell wall. For instance, MSMEG6281, a peptidoglycan (PG) amidase, is essential in maintaining cell wall integrity [10]. And succinate dehydrogenase (Sdh), encoded by MSMEG0416-MSMEG0420 and MSMEG1672-MSMEG1669 in Ms, is a pivotal respiratory enzyme that facilitates the oxidation of succinate to fumarate in the cytoplasm and concomitantly reduces quinone to quinol in the membrane [11]. In addition, mycobacteria must retain their complex cell envelope as they grow.

The cell envelope plays a crucial role in mycobacteria pathogenesis, as most virulence factors are exposed to this structure [12]. Furthermore, changes in the cell envelope can also affect the proper transmission of mechanical signals within the local cell membrane, and this malfunction often leads to the development of host diseases [13,14]. For instance, Mtb lipoproteins elicit TLR2-mediated signaling in macrophages, which hinders the IFN-γ-induced expression of MHC-II molecules [15,16]. Meanwhile, the secretion system is also localized within the region, facilitating the extracellular release of secreted proteins [17,18]. Recent evidence implied that mycobacteria develop novel and special secretion systems to transport extracellular proteins through their hydrophobic and impermeable cell walls [19]. Strikingly, mycobacterial genomes encode up to five transport systems [20]. A series of studies have revealed ESX systems in bacterial survival and pathogenicity during infection with M. tuberculosis [21]. ESX proteins mediate toxin secretion, and EsxF is accessible on the bacterial cell surface. ESX systems might be applied to develop novel strategies for treating and preventing disease [22]. Although the functional implications of membrane proteins in mycobacteria remain unknown, their indispensable role in the immune enigmatic cannot be denied.

Bioinformatics methods are used to align homologous membrane proteins with unknown functions in Ms and Mtb, aiming to explore the role of these proteins in mycobacteria growth [23]. From the results, we noticed that MSMEG5257 is a channel protein of unknown function annotated as the hemolysin III families in Ms. Recent studies have also pinpointed that *Rv1085c*, the homologous proteins of *msmeg5257* in Mtb, plays a role in iron uptake, forming the ESX-4 secretion system [24,25]. Based on this ground, we hypothesized that *msmeg5257* might be involved in the process of Ms growth.

In this study, we constructed Δ*msmeg5257* strains through CRISPR/Cas9 to validate our hypothesis. The utilization of Clustered Regularly Interspaced Short Palindromic Repeat (CRISPR)–CRISPR-associated protein (Cas) genome editing has demonstrated its remarkable efficacy as a potent genetic tool across eukaryotic and bacterial cells [26]. The CRISPR/Cas9 system induces a Cas9-mediated double-strand break (DSB) that could be guided by a chimeric single guide RNA (sgRNA). Recognition and cleavage of target DNA by CRISPR/Cas require a short DNA sequence adjacent to the protospacer, known as the protospacer adjacent motif (PAM). The DSBs can be repaired through the nonhomologous end-joining (NHEJ) repair pathway, resulting in imprecise genome editing. The NHEJ pathway repairs the DSBs by inducing indels at the cleavage site, leading to disruption of the targeted gene [27]. Meanwhile, we utilized RNA sequencing, an LC-MS/MS analysis, and a growth curve to explore the functions of *msmeg5257* in Ms growth. We observed that the deletion of *msmeg5257* resulted in the inhibition of ABC transporters and metabolic processes in the cell envelope, leading to an increased growth rate of Ms.

## 2. Materials and Methods

### 2.1. Bacterial Strains, Primers, and Media

*M. smegmatis* mc^2^155 and *E. coli* DH5α were used in the study. Mycobacteria strains were grown in Middlebrook 7H9 medium or Middlebrook 7H10 medium containing 0.2% glycerol and 0.05% Tween 80 at 37 °C. *E. coli* DH5α was cultured in LB medium at 37 °C with 200 rpm shaking. If required, hygromycin (50 μg/mL), kanamycin (50 μg/mL), or anhydrotetracycline (100 ng/mL) should be added to the medium. The antibiotics were procured from Sigma-Aldrich, Shanghai, China, Merck. The medium was procured from BD. The primers used in the study are shown in Supplementary Table S1.

### 2.2. Construction of msmeg5257 Mutant Strains

The *msmeg5257* gene fragment was cloned to the pSUM-Kan-MCS2 plasmid for the overexpression of *msmeg5257*. The sgRNAs (CCCAGTTCTTCGGAAAACCA) were designed from Guide Design Resources (https://zlab.bio/guide-design-resources, accessed on 27 December 2020). The fragment of the sgRNA sequence and pRH2521 plasmid were digested with BbsI enzyme (NEB) for appropriate sticky end overhangs and annealed and ligated with T4 ligase (NEB). We used DNA Sanger sequencing to confirm the success of all construct cloning. The pRH2502-Cas9 plasmid was generated through the substitution of Ala at position 10 with Asp and Ala 840 at the position with His in the dCas9 amino acid (AA) sequence of pRH2502-dCas9. We used DNA Sanger sequencing to confirm the success of all construct cloning. The primers used in the study are shown in Supplementary Table S1. Then, CRISPR/Cas9 (pRH2502-Cas9 and sgRNA plasmids) were electroporated into *M. smegmat* mc^2^155 based on electroporation at 2500 V, 1000 Ω, and 25 μF with a Gene Pulser X cell electroporation system (Bio-Rad, Laboratories, Shanghai, China). The Δ*msmeg5257* strains were complemented by reintroducing a wild-type (WT) copy of *msmeg5257* expressed from its native promoter integrated at Ms. Ms was recovered in Middlebrook 7H9 medium, which contained 0.2% glycerol and 0.05% Tween 80, for 2 h at 37 °C with 200 rpm shaking, then supplemented with hygromycin (50 μg/mL), kanamycin (50 μg/mL) and anhydrotetracycline (100 ng/mL) for an OD_600_ of 0.8. Then, the cells were plated on 7H10 agar supplemented with antibiotics. All ligases were procured from NEB (Beijing, China).

The genomic DNA sequence was extracted using the cetyltrimethylammonium bromide (CTAB) method, as previously described [28]. Purified DNA samples were subjected to whole-genome sequencing using the Illumina HiSeq PE150 technique by Sangon Biotech (Shanghai, China).

### 2.3. Western Blotting

The samples were resuspended in the SDS loading buffer and heated at 100 °C for 10 min. The eluted proteins were applied to SDS-polyacrylamide gels and transferred to the PVDF membrane. The membranes were blocked with 2% bovine serum albumin (BSA) for 2 h. Then, immunoblotting was performed with antibodies for 1 h, and the membranes were washed three times with TBST (20 mM Tris-HCl [pH 7.6], 0.15 M sodium chloride, and 0.1% Tween 20) and then incubated with secondary antibodies for 1 h. Finally, the membranes were washed three times with TBST. Blots were developed by chemiluminescence using Pierce ECL Western Blotting Substrate with the ChemidocTM Touch imaging system (Bio-Rad Laboratories, Shanghai, China) and analyzed with Image Lab software (Beta 3) (Version3, Bio-Rad, Laboratories, Shanghai, China). The PVDF membrane was purchased from Millipore (Merck, Shanghai, China). The Pierce ECL Western Blotting Substrate was purchased from Thermo Fisher Scientific (Thermo Fisher Scientific Inc., Beijing, China). At Sangon Biotech (Shanghai, China), polyclonal anti-MSMEG5257 antibody (rabbit) was generated by immunization rabbits with the peptide antigen sequence of MSMEG5257 (NWTSVTARKWMKR), and ELISA determined the titers of anti-MSMEG5257.

### 2.4. RNA-Seq Experiments and Real-Time PCR

Mutant strains were pre-grown on Middlebrook 7H9 medium. Total RNA was isolated from mutant strains using TRIzol at different times. The cultures were extracted as per the protocol. Firstly, the cultures were harvested, washed, and resuspended in 1 mL of TRIzol at 4 °C. Subsequently, the mixture was extracted with 200 μL of chloroform at RT for 5 min, and the supernatant was precipitated with the same volume of isopropanol. Then, the precipitation was washed twice with 70% ethanol anhydrous. Finally, RNA was eluted in nuclease-free water. RNA was sequenced on an Illumina HiSeq at Sangon Biotech (Shanghai, China). We used the limma package installed in the R programming language for RNA-seq data analysis to identify differently expressed genes (|log_2_ Fold Change| > 1, *p* values < 0.05) in mutant strains. Sequenced and quality-controlled raw reads were mapped to *M. smegmatis* mc^2^155. Trinity was used to perform a gene-level summarization of mapped reads. The TMM (trimmed mean of M-values) was normalized for the counted reads of each gene. Then, DEGseq was used for the statistical analysis of differential gene expression. To conduct a statistical analysis of pathway enrichment, we screened the collection significantly at the following database: Kyoto Encyclopedia of Genes and Genomes (KEGG) (https://www.kegg.jp, accessed on 20 June 2022).

The extracted RNA was converted into cDNA using 100 ng of RNA and a RevertAid RT Reverse Transcription Kit. RT-PCR reactions were performed in 96-well plates using GC SYBR Green Master Mix. Signals were quantified by the 2^−ΔΔCt^ method. The primers used in the study are shown in Supplementary Table S1. All chemicals and the RevertAid RT Reverse Transcription Kit were procured from Thermo Fisher Scientific (Thermo Fisher Scientific Inc., Beijing, China). The GC SYBR Green Master Mix was procured from TAKARA (Takara Bio Inc., Beijing, China).

### 2.5. Drug Susceptibility Testing

The minimum inhibitory concentration (MIC) refers to the antibiotic concentration at which observable growth of microorganisms is inhibited, and it represents the lowest level of inhibition. Ms_WT and Ms_Δ*msmeg5257* were pre-grown in 5 mL of Middlebrook 7H9 medium until an OD_600_ of 0.8. Cells were harvested by centrifugation at 3000× *g* for 10 min, and the bacterial pellets were resuspended in 5 mL of 0.9% NaCl. The resuspended 2 mL mixture was subsequently transferred to an ultrasonic tube and adjusted to a bacterial suspension concentration of 0.5 Ω using the ultrasonic dispersion counter. The 100 μL 0.5 Ω bacterial suspension was aspirated into 9.9 mL of Middlebrook 7H9 medium and vortexed for 30 s. Then, the 10 mL bacterial suspension was inoculated into 96-well cell culture plates, which had added different concentrations of drugs, with 100 μL per well. The plates were incubated at 37 °C for three days in an incubator, followed by a result analysis using the Thermo Scientific Sensititre Vizion Digital MIC Viewing System. The drug name and concentration are shown in Supplementary Table S2. The sensititre susceptibility plates (96-well cell culture plates containing various drug concentrations) were procured from Thermo Scientific (UKMYC6, Thermo Fisher Scientific Inc., Beijing, China).

### 2.6. Growth of msmeg5257 Mutant Strains in Iron-Depleted Agar

The ddH_2_O was stirred with Chelex 100 resin at 4 °C overnight to eliminate residual iron. Subsequently, the Chelex 100-treated solution was sterilized by filtration through a 0.22 μm pore-size filter (Millipore, Merck, Shanghai, China). Then, 2.5 g/L disodium hydrogen phosphate, 1 g/L monopotassium phosphate, 5 mM ammonium sulfate, 300 μM sodium citrate, 200 μM magnesium sulfate, 5 μM calcium chloride, 5 μM zinc acetate, 5 μM copper sulfate, 0.2% glycerol, and 15 g/L agar were added to the iron-depleted medium (the medium prepared in this manner exhibited a significant reduction in iron levels, estimated to be 0.3 μM based on ferrozine iron assays [29]). If required, the medium was supplemented with 50 μM hemin. The cultures were adjusted to an OD_600_ of 0.1, and 5 μL aliquots of undiluted bacteria or dilutions of 10^1^, 10^2^, and 10^3^ were spotted on iron-depleted agar plates containing various concentrations of hemin (1 or 50 μM). Plates were photographed at 3 days. Three independent experiments were conducted for each strain. The Chelex 100 resin was procured from Shanghai Macklin Biochemical Technology (Shanghai, China). The hemin was procured from Beyotime Biotechnology (Shanghai, China).

### 2.7. Subcellular Fractionation of msmeg5257

Ms_WT, Ms_Δ*msmeg5257*, Ms_OE-*msmeg5257*, and Ms_C-Δ*msmeg5257* were pre-grown in 200 mL of Middlebrook 7H9 medium containing 0.2% glycerol and 0.05% Tween 80 until an OD_600_ of 0.8. If necessary, kanamycin (50 μg/mL) was supplemented to the medium. The culture was harvested by centrifugation at 5000× *g* for 10 min at 4 °C, and we washed pellets once with PBS. The bacterial pellets were resuspended in 10–13 mL of PBS containing PMSF (1 mM), DNase (0.6 μg/mL), and RNase (0.6 μg/mL). Next, the mixture was sonicated with 15 × 10 s pulses, with 10 s rests between the pulses for 30 min. The lysates were pelleted at 3000× *g* for 20 min at 4 °C to remove unlysed cells and cell debris. The supernatant was removed to a new tube and centrifuged at 27,000× *g* for 30 min at 4 °C to obtain the cell wall pellet. Simultaneously, the supernatant from the last step was centrifuged at 100,000× *g* for 2 h at 4 °C to separate the cell membrane and cytosol fractions. Ultimately, the pellets from the cell wall and cell membrane were washed once with PBS and resuspended in the SDS loading buffer.

### 2.8. Label-Free Mass Spectrometry Protein Quantification

Ms_WT, Ms_Δ*msmeg5257*, and Ms_C-Δ*msmeg5257* were pre-grown in 200 mL of Middlebrook 7H9 medium containing 0.2% glycerol and 0.05% Tween 80 until an OD_600_ of 0.8. If necessary, kanamycin (50 μg/mL) was supplemented to the medium. The cell wall, cytomembrane, and cytoplasm were isolated using ultra-fast centrifugation. Then, lysis solution (8 M Urea/100 mM Tris-HCl) was added to those samples, followed by sonication in a water bath. The dithiothreitol (DTT) was added and incubated at 37 °C for 1 h. Subsequently, iodoacetamide (IAA) was added, and the sulfhydryl group was blocked by an alkylation reaction at room temperature in the dark. Protein concentration was determined using the Bradford method. Tris-HCl (100 mM) solution was added to the reduced and alkylated samples, the urea concentration was diluted to less than 2 M, trypsin was added according to the mass ratio of enzyme to the protein of 1:50, and the enzyme was incubated at 37 °C with shaking overnight for digestion. TFA was added to terminate digestion the next day, and the supernatant was removed for Sep-Pak C18 desalting. They were drained and frozen at −20 °C until use. Mass spectrometry protein quantification was sequenced by Sangon Biotech (Shanghai, China). Mass spectrometry was performed using a timsTOF Pro instrument from Bruker. Sample injection and separation were performed using an UltiMate 3000 RSLCnano liquid chromatography system that was online coupled to the mass spectrometer. MaxQuant (V1.6.6) software retrieved the mass spectrum data, and the database retrieval algorithm used was Andromeda. The database used for the search was the proteome reference database of *M. smegmatis* mc^2^155 in Uniprot.

### 2.9. Growth of msmeg5257 Mutant Strains in Middlebrook 7H9 Media

Ms_WT, Ms_*vec*, Ms_Δ*msmeg5257*, Ms_OE-*msmeg5257*, and Ms_C-Δ*msmeg5257* were pre-grown in Middlebrook 7H9 medium containing 0.2% glycerol and 0.05% Tween 80 at 37 °C with shaking at 200 rpm until an OD_600_ of 0.8. The cultures were inoculated into 200 mL of Middlebrook 7H9 medium containing 0.2% glycerol and 0.05% Tween 80 at an initial OD_600_ of 0.01. If necessary, kanamycin (50 μg/mL) was supplemented to the medium. The 200 μL culture was transferred to a new 96-well plate, and absorbance at OD_600_ was measured in a Bio-Rad iMark microplate reader. All experiments were performed in triplicate. The cell growth was measured for 48 h, and the OD_600_ was determined every 12 h.

### 2.10. Statistical Analysis

We performed all assays independently at least three times. We expressed all data as the mean ± standard deviation (SD). Two-tailed Student’s *t*-tests were used for all statistical analyses. GraphPad Prism version 9 (GraphPad Software Inc., San Diego, CA, USA) was used for the statistical analysis and generation of graphs. Consider *p* values < 0.05 as there being a significant difference.

## 3. Results

### 3.1. Construction of msmeg5257 Deletion Strains

**Figure 1:**
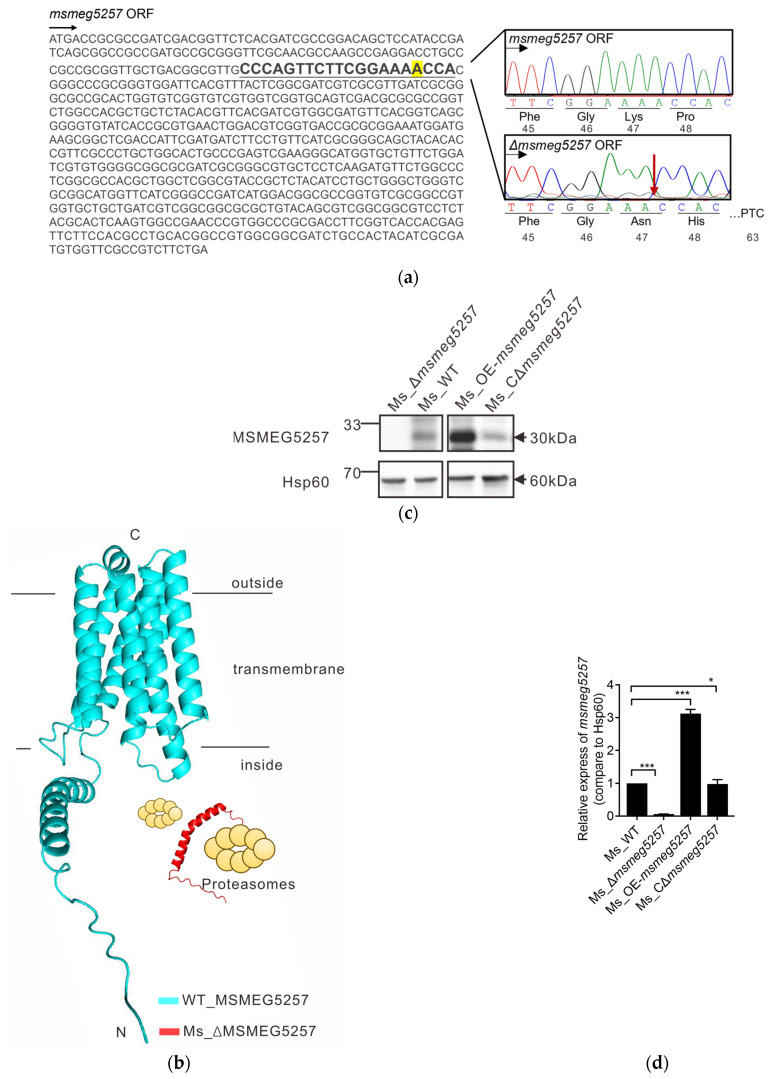
Construction of *msmeg5257* mutant strains. (**a**) The DNA sequence of the wild-type *msmeg5257* ORF (left) and the deletion of *msmeg5257* at position 141 A (highlighted in the sgRNAs sequence) resulted in a modification of its ORF amino acid sequence (right, red arrow). Red arrow, Location of base deletion. (**b**) The premature termination of the *msmeg5257* translation was caused by the deletion at position 141 A. The newly synthesized misfolded 45-amino acid protein will be subject to degradation by bacterial proteases [32]. ORF, open reading frame. (**c**,**d**) Western blotting analysis of the expression of MSMEG5257 protein (**c**) and quantitative image analysis of Western blotting (**d**) on *msmeg5257* mutant strains. ***, *p* values < 0.001 as a very significant difference; *, *p* values < 0.05 as a significant difference.

The commonly employed induction antibiotic for constructing mycobacterial deletion systems is tetracycline [30]. To eliminate the interference effect of tetracycline on the phenotype of mutant strains, we developed a clustered regularly interspaced short palindromic repeats-Cas9 (CRISPR/Cas9) system based on CRISPR/dCas9 [31]. Firstly, we successfully constructed the pRH2502-Cas9 plasmid (Supplementary Figure S1a). Then, we used clustered regularly interspaced short palindromic repeats-Cas9 (CRISPR/Cas9) to knock out *msmeg5257* in Ms. The PCR sequencing analysis revealed that deletion at position 141 A in the *msmeg5257* gene sequence changed the amino acid sequence (Figure 1a). The simultaneous application of whole-genome sequencing analysis yielded identical findings (Supplementary Figure S1b). Our AlphaFold prediction suggested that the mutant structure will result in the premature termination of the expression of *msmeg5257* (Figure 1b red). Secondly, we overexpressed *msmeg5257* in Ms using the pSUM-Kan-MCS2 plasmid, and the control group was electroporated with the pSUM-Kan-MCS2 plasmid. Finally, the deletion and overexpression of *msmeg5257* were confirmed by probing the *msmeg5257* protein in the whole cell lysate by Western blotting and a quantitative image analysis with Hsp60 as a loading control. As expected, the quantitative image analysis of Western blotting showed that MSMEG5257 was detected in the whole cell lysate of Ms_WT (Figure 1c,d). Meanwhile, the results showed that the expression of MSMEG5257 protein was upregulated approximately threefold in Ms_OE-*msmeg5257* compared with the wild type (Figure 1c,d). Also, the expression of the complemented MSMEG5257 was validated in Ms_C-Δ*msmeg5257* (Figure 1c,d).

### 3.2. KEGG Enrichment Analysis of RNA Data in msmeg5257 Deletion Strains

**Figure 2:**
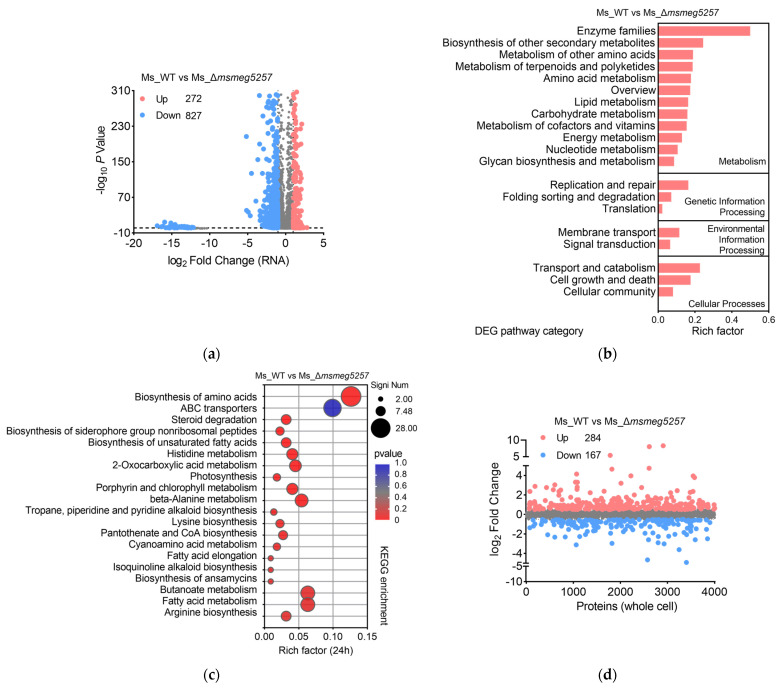
The transcriptome of Δ*msmeg5257* strains was subjected to a KEGG pathway enrichment analysis. (**a**) The volcano plot depicts the differential gene transcription in Δ*msmeg5257* strains compared with wild-type strains. The upregulated genes are highlighted in red; the downregulated genes are highlighted in blue. |log_2_ Fold Change| > 1 and *p* values < 0.05 are noted as a difference expression gene (DEG). (**b**) The histogram analysis of DEG pathway categories in Δ*msmeg5257* strains. (**c**) A KEGG enrichment analysis was performed to investigate the DEGs of Δ*msmeg5257* strains. (**d**) Scatterplots illustrating the differential protein expression in the whole-cell lysis of Δ*msmeg5257* strains compared with wild-type strains. The upregulated proteins are highlighted in red; the downregulated proteins are highlighted in blue. |log_2_ Fold Change| > 0.5 is noted as a differentially expression protein. Up, upregulated; Down, downregulated.

To investigate the impact of deletion *msmeg5257* during the mid-log phase of Ms growth, we categorized the growth curve of Ms into early (0–12 h), middle (13–36 h), late (37–48 h), and very late (>48 h) stages (Supplementary Figure S1). Then, we performed RNA sequencing at 24 h on Ms_Δ*msmeg5257*, Ms_C-Δ*msmeg5257*, or Ms_WT based on the growth curve of Ms (Supplementary Figure S1). The |log_2_ Fold Change| > 1 and *p* values < 0.05 were considered differently expressed genes (DEG) for RNA analysis. The results showed that 75.3% (827/1099) of DEGs were downregulated in Ms_Δ*msmeg5257* (Figure 2a). The pathway categories analysis revealed significant alterations in the DEGs associated with metabolism, genetic information processing, environmental information processing, and cellular processes in Ms_Δ*msmeg5257* compared with Ms_WT (Figure 2b).

Among them, the subgroups of enzyme families, replication and repair, membrane transport, and transport and catabolism exhibited the most significant changes in the respective groups (Figure 2b). The KEGG pathway analysis revealed consistent results, indicating significant enrichment of the biosynthesis of amino acids, ABC transporters, and fatty acid metabolism pathways in Ms_Δ*msmeg5257* (Figure 2c). In the meantime, the deletion of *msmeg5257* significantly impacted metabolic pathways like arginine biosynthesis, histidine metabolism, and lysine biosynthesis based on the KEGG analysis (Figure 2c). Those results suggested that the deletion of *msmeg5257* affects the metabolic processes for Ms on the transcriptional level.

### 3.3. The Correlative Analysis of LC-MS/MS and RNA Data in msmeg5257 Deletion Strains

**Figure 3:**
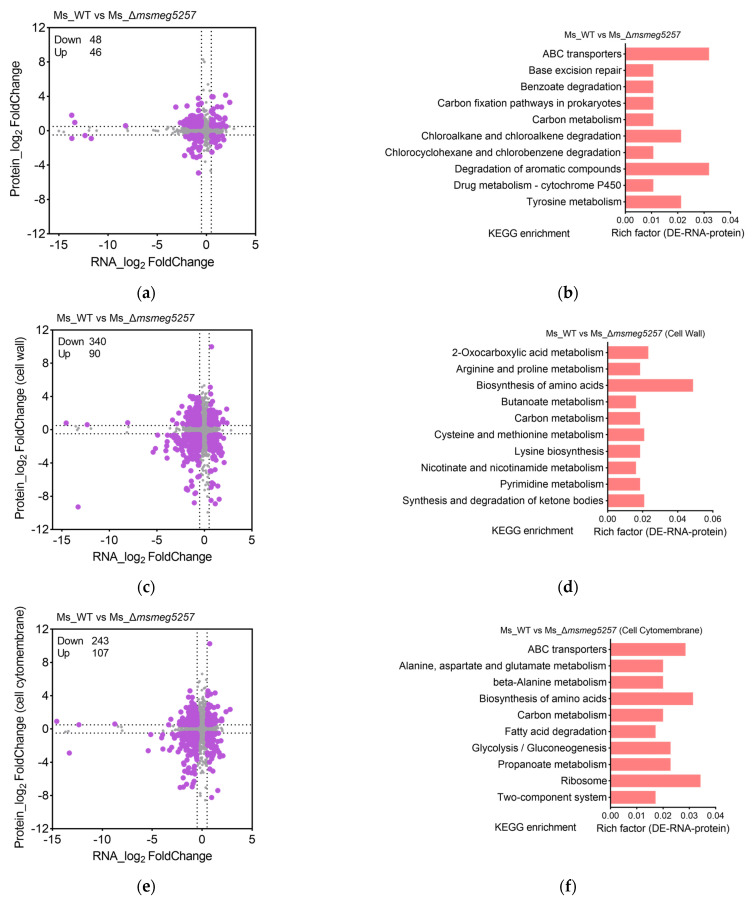

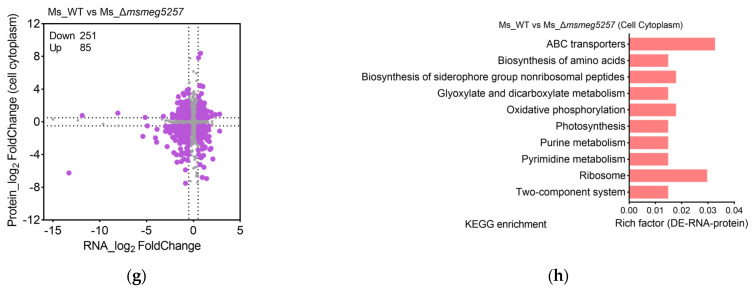
The correlative analysis between RNA data and MS data in subcellular fractions of Δ*msmeg5257* strains, including the whole cell (**a**), cell wall (**c**), cytomembrane (**e**), and cytoplasm (**g**). The correlative analysis of RNA data and MS data was conducted to identify the KEGG enrichment pathways in the whole cell (**b**), cell wall (**d**), cytomembrane (**f**), and cytoplasm (**h**) of Δ*msmeg5257* strains, respectively. DE, differently expressed; Up, upregulated; Down, downregulated.

To explore the mechanism of *msmeg5257* in material transport, we performed proteomic profiling on the deletion mutant strains, grown until the mid-log phase, by liquid chromatography–tandem mass spectrometry (LC-MS/MS). |log_2_ Fold Change| > 0.5 was considered a differentially expressed protein (DEP) for proteome analysis. Compared with Ms_WT, Ms_Δ*msmeg5257* has 63% (284/451) of DEPs upregulated and 37% (167/451) of DEPs downregulated (Figure 1d). However, the correlative analysis between RNAseq and MS data revealed a significant association among 94 co-differently expressed RNA and proteins (DRPs) (Figure 3a). The subsequent KEGG enrichment analysis revealed that the top three pathways were ABC transporters, degradation of aromatic compounds, and tyrosine metabolism (Figure 3b).

### 3.4. Deletion of msmeg5257 Inhibits Expression in ABC Transporters of Cytomembrane

**Figure 4:**
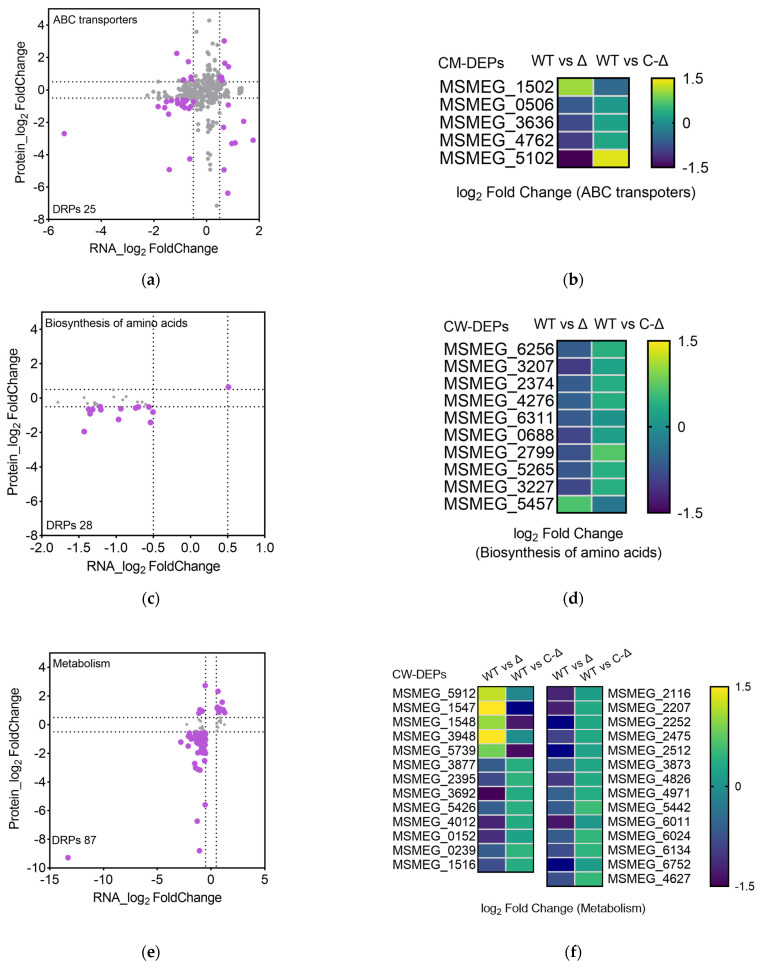
The correlative analysis of RNA data and MS data in Δ*msmeg5257* strains for ABC transporters (**a**), biosynthesis of amino acids (**c**), and metabolism (**e**). (**b**) The heat map illustrates the DEPs associated with ABC transporters in the cytomembrane of both the deletion and complementation strains of *msmeg5257*. (**d**,**f**) The heat map illustrates the DEPs associated with the biosynthesis of amino acids (**d**) and metabolism (**f**) in the cell wall of both the deletion and complementation strains of *msmeg5257*. DRPs, differently expressed-RNA-proteins; DEPs, differentially expressed proteins; CM, cytomembrane; CW, cell wall; WT, Ms_WT; Δ, Ms_Δ*msmeg5257*; C-Δ, Ms_C-Δ*msmeg5257*.

To assess the impact of *msmeg5257* deletion on ABC transporters, we performed subcellular fractions proteomic profiling on the mutant strains grown until the mid-log phase. The |log_2_ Fold Change| > 0.5 was considered as DEPs for the proteome analysis. The DRPs analysis results from Ms_Δ*msmeg5257* revealed that 90 DRPs were increased and 340 were decreased in the cell wall (Figure 3c); 243 DRPs were increased and 107 were decreased in the cytomembrane (Figure 3e); 251 DRPs were increased and 85 were decreased in the cytoplasm (Figure 3g). The KEGG enrichment analysis of different subcellular fractions was also conducted for the DRPs. The analysis results showed that the DRPs of the biosynthesis of amino acids were enriched in the cell wall and cytomembrane (Figure 3d,f); the DRPs of the ribosome were enriched in the cytomembrane and cytoplasm (Figure 3f,h). The DRPs of the ABC transporters were enriched in cytomembrane and cytoplasm (Figure 3f,h), which included 25 DRPs (Figure 4a). The findings imply that the elimination of *msmeg5257* could potentially influence the growth of Ms.

The comparative analysis of DRPs between Ms_Δ*msmeg5257* and Ms_C-Δ*msmeg5257* showed that the deletion of *msmeg5257* resulted in under-expressed ABC transporters, such as MSMEG0506, MSMEG3636, MSMEG4762, and MSMEG5102 in the cytomembrane (Figure 4b). The drug susceptibility of the mutant strain was further investigated to investigate the impact of deletion *msmeg5257* on ABC transporters. The results showed that the deletion of *msmeg5257* led to a slight decrease in resistance to bedaquiline and clofazimine in Ms (Supplementary Table S3), and there were no changes in other drugs.

We also analyzed the biosynthesis of amino acids, which included 28 DRPs (Figure 4c). The results showed that MSMEG6256, MSMEG3207, MSMEG2374, MSMEG4276, MSMEG6311, MSMEG0688, MSMEG2799, MSMEG5265, and MSMEG3227 were decreased in the cell wall of Ms_Δ*msmeg5257* (Figure 4d). Then, we analyzed 87 metabolism-related DRPs, and the results showed that MSMEG1547 and MSMEG3948 were decreased in the cell wall of Ms_Δ*msmeg5257* (Figure 4e,f). The expression of these proteins exhibited an inverse relationship in Ms_C-Δ*msmeg5257* (Figure 4b,d,f).

### 3.5. MSMEG5257 Localizes in the Cytomembrane of Ms

**Figure 5:**
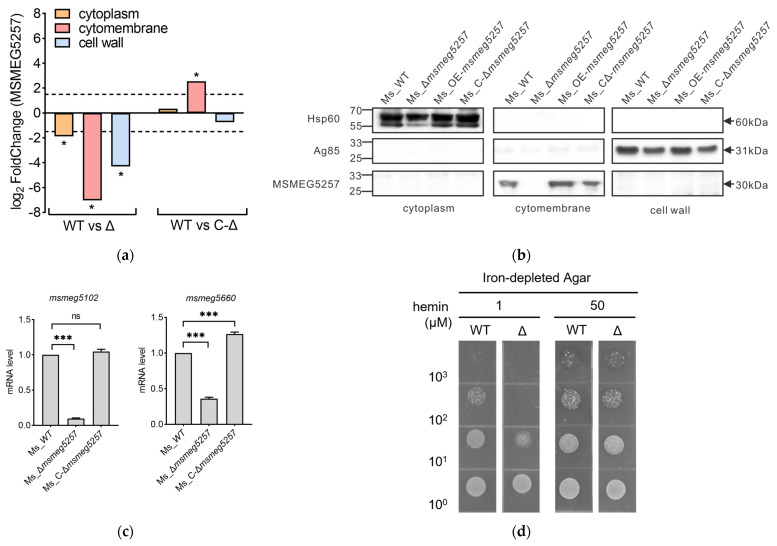
MEMEG5257 localizes within the cytomembrane of Ms and has a role in heme-iron acquisition. (**a**) LC-MS/MS analysis showed that MSMEG5257 was localized in the cytomembrane. WT, Ms_WT; Δ, Ms_Δ*msmeg5257*; C-Δ, Ms_C-Δ*msmeg5257*; *, |log_2_ Fold Change| > 1.5 as a significant difference. (**b**) The Western blotting results showed the subcellular localization of MSMEG5257 proteins in mutant strains. Hsp60 serves as a cytoplasm control, Ag85 as a cell wall control. (**c**) The analysis of alterations in the mRNA transcription levels of *msmeg5102* and *msmeg5660* in deletion strains was conducted by RT-PCR. ***, *p* values < 0.001 as a very significant difference; ns, no difference. (**d**) The growth of Ms on iron-depleted agar plates was impeded upon deletion of *msmeg5257*. Bacterial cultures were standardized to an OD_600_ of 0.1, and undiluted bacteria (10^0^) or dilutions (10^1^, 10^2^, and 10^3^) were applied as spots on agar with varying concentrations of hemin (1 or 50 μM). WT, Ms_WT; Δ, Ms_Δ*msmeg5257*.

We hypothesize that the alterations in ABC transporters induced by *msmeg5257* are presumed to be linked with its intracellular localization. Meanwhile, The LC-MS/MS analysis showed that the level of MSMEG5257 protein was significantly expressed in the cytomembrane (Figure 5a). To validate these observations at the subcellular level, we conducted ultracentrifugation and Western blotting to analyze the localization of MSMEG5257. The results demonstrated that the subcellular localization of MSMEG5257 in Ms_WT, Ms_OE-*msmeg5257*, or Ms_C-Δ*msmeg5257* was unequivocally confirmed to be in the cytomembrane (Figure 5b). Subcellular fractionation of the mutant strains showed a clear separation of cytoplasm versus cell wall fractions, as indicated by the marker proteins Hsp60 and Ag85 (Figure 5b).

### 3.6. Deletion of msmeg5257 Inhibits the Growth of Ms in Iron-Depleted Media

Based on the correlative analysis of DRPs and subcellular localization results, we selected genes involved in the biosynthesis of the siderophore group nonribosomal and quantified their mRNA levels using RT-PCR to validate our RNAseq data. The results revealed that, except for *msmeg4509*, *msmeg4510*, and *msmeg4512*, the transcript levels of all selected genes were downregulated in Ms_Δ*msmeg5257* (Supplementary Figure S3 and Figure 5c). Among these genes, *msmeg5102* and *msmeg5660* are classified as ABC transporter ATP-binding proteins (Figure 5c), while *msmeg5102* belongs to DRP (Figure 4b). To assess the impact of Ms_Δ*msmeg5257* on the heme-iron acquisition (HIA) phenotype, we examined the growth of Ms_Δ*msmeg5257* on the iron-depleted medium. We observed that wild-type strains at dilutions of 10^3^ exhibited growth even at hemin concentrations as low as 1 μM, whereas Δ*msmeg5257* strains displayed slightly visible growth only when diluted to a concentration of 10^1^ and barely visible growth at dilutions of 10^2^ and 10^3^ (Figure 5d). When the heme concentration was 50 μM, no significant difference was observed in the wild-type or Δ*msmeg5257* strains (Figure 5d).

### 3.7. Deletion of msmeg5257 Increases the Growth Rate of Ms In Vitro

**Figure 6:**
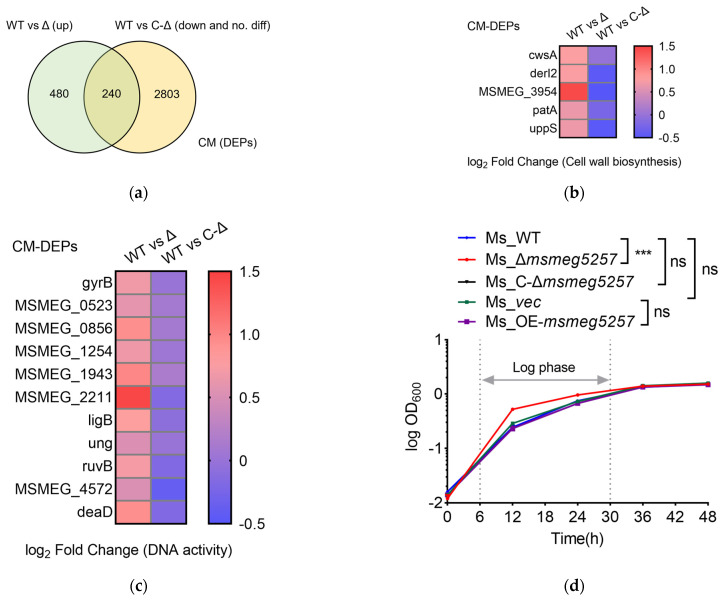
The growth curve of *msmeg5257* mutant strains. (**a**) Venn diagrams showing the differential expression proteins (DEPs) in the upregulated group of Ms_Δ*msmeg5257*, the downregulated group of Ms_C-Δ*msmeg5257*, and the non-differentiated group of Ms_C-Δ*msmeg5257* in the cytomembrane. WT, Ms_WT; Δ, Ms_Δ*msmeg5257*; C-Δ, Ms_C-Δ*msmeg5257*; up, upregulated; down, downregulated. (**b**) The heat map illustrates the DEPs associated with the cell wall biosynthesis processes in the cytomembrane of both the deletion and complementation strains of *msmeg5257*. (**c**) The heat map illustrates the DEPs associated with the DNA activity in the cytomembrane of both the deletion and complementation strains of *msmeg5257*. (**d**) The growth curve of *msmeg5257* mutant strains in vitro. The results demonstrated a significant increase in the growth rate of Ms during the logarithmic phase (6 to 36 h) following the deletion of *msmeg5257.* ***, *p* values < 0.001 as a very significant difference; ns, no difference, no statistical significance; CM, cytomembrane; DEPs, differently expressed proteins; WT, Ms_WT; Δ, Ms_Δ*msmeg5257*; C-Δ, Ms_C-Δ*msmeg5257*; up, upregulated; down, downregulated.

To further investigate the impact of Δ*msmeg5257* on Ms growth, we conducted LC-MS/MS to examine the growth-related proteins in the cytomembrane component of mutant strains. The results showed that 240 DEPs were increased in the cytomembrane component of Ms_Δ*msmeg5257* (Figure 6a). Among them, the expression levels of key genes involved in cell wall biosynthesis processes, namely cwsA, derl2, patA, uppS, and MSMEG3954, were significantly upregulated in Ms_Δ*msmeg5257* (Figure 6b). Furthermore, the KEGG analysis revealed that the ribosome was enriched in the cytomembrane (Figure 3f). We further investigated the impact of the DNA helicase activity and DNA replication. The results showed that the expression levels of key proteins involved in DNA helicase activity or DNA replication, namely gyrB, MSMEG1943, MSMEG2211, ruvB, MSMEG4572, and deaD, were also found to be upregulated in Ms_Δ*msmeg5257* (Figure 6c). 

Therefore, we hypothesized that *msmeg5257* might be associated with the growth rate of Ms. The results demonstrated a significant increase in the growth rate of Ms during the logarithmic phase (6 to 36 h) following the deletion of *msmeg5257* (Figure 6d). In contrast, we have observed that the growth of *msmeg5257* overexpression was similar to that observed for the empty vector strains (Figure 6d). The growth rate of the *msmeg5257* deletion mutant was fully restored to wild-type levels by complementation with wild-type *msmeg5257* in the logarithmic growth phase (Figure 6d). The observations confirm that the modified growth pattern of the deletion strains can be attributed to the deletion of *msmeg5257*.

## 4. Discussion

The unique cell envelope of mycobacteria enables them to defend against host immune attacks and prevent antibiotic penetration [23,33]. Meanwhile, many bacterial proteins involved in various cellular functions were localized at distinct focal regions on the bacterial membrane [34], such as the MmpL family, ABC transporter family, and SMR family [24]. Among the prescribed drugs for TB treatment, 60–70% of them target onto membrane proteins [35,36]. Therefore, the bacterial membrane proteins cannot be accurately expressed; this could impact the growth and pathogenic processes of bacteria, and sometimes, it can be fatal [37,38].

Our study highlights the Δ*msmeg5257* on regulatory control over ABC transporters and metabolic processes, resulting in an increased growth rate in Ms. The deletion of *msmeg5257* caused significant changes in the transcript levels of genes related to metabolism and membrane transport. This finding suggests that the expression of membrane-related proteins is affected following the deletion of *msmeg5257*, which leads to the cell envelope’s remodeling. The expression of certain enzymes involved in cell wall synthesis was downregulated. For instance, MSMEG6256, for which its homologous protein Asd (Rv3708c) in Mtb is the enzyme that lies at the first branch point in the cell wall component diaminopimelate (DAP) that serves as an example [39]. MSMEG3207, with its homologous protein HisB, which is encoded via open reading frame Rv1601, functions as an imidazoleglycerol-phosphate dehydratase in the histidine biosynthetic pathway of Mtb [40]. Likewise, *msmeg5257* can affect the material transport and energy metabolism, such as those related to arginine, fatty acids, and glycolysis. Arginine utilization, fatty acid metabolism, and glycolysis contribute to thallus protein synthesis and expression [41]. The expression of the biosynthesis of mycolic acid was enhanced in Ms_Δ*msmeg5257*. Mycolic acid, a long-chain fatty acid found in the cell walls of mycobacteria, exhibits anti-acidic and antioxidant properties and plays a crucial role in bacterial growth and survival [42]. In particular, mycolic acids play a crucial role in determining fluidity and contribute to the impermeability of the mycobacteria cell wall [43]. The suggestion is that the permeability of the cell envelope may also change after the deletion of *msmeg5257*, and this was also confirmed via the subsequent correlative analysis results of LC-MS/MS and RNA data, such as the suppression of ABC transporter proteins in the cytomembrane.

ABC transporters are involved in a variety of important transportation processes in organisms, which necessitate the participation of functional activity from both nucleotide-binding domains (NBDs) and transmembrane domains (TMDs) [44,45]. The TMDs contribute to mycobacterial pathogenesis by secreting virulence factors and promoting antibiotic efflux [46]. For instance, the deletion of MSMEG3763 enhances the susceptibility of Ms relative to antimicrobial drugs, which are classified as transmembrane polypeptides belonging to the ABC efflux pump in the MSMEG3762-MSMEG3763-MSMEG3765 operon of Ms [47]. This effect was probably due to ABC transporters facilitating the translocation of lipids from plastids to the endoplasmic reticulum through energy produced via ATP hydrolysis, thereby contributing to the maintenance of stability in cell membrane lipid content [48]. Additionally, ABC transporters participate in lipid translocation between inner and outer membranes, regulating lipid asymmetry and subsequently controlling cell membrane permeability [49,50]. The increased susceptibility to bedaquiline and clofazimine following the deletion of *msmeg5257* may be attributed to the downregulation of ABC transporter ATP-binding proteins, such as MSMEG1502 and MSMEG5102, as well as membrane remodeling. The inhibition of ATP production by bedaquiline is achieved via the direct targeting of the mycobacterial ATP synthase [51]. Clofazimine can also lead to the membrane destabilization of mycobacteria [52]. The implication is that *msmeg5257* may play a role in the mechanism of mycobacterial resistance relative to bedaquiline and clofazimine; however, additional data are required to substantiate this hypothesis.

Several studies have reported that ABC transporter components are involved in the uptake of metals, which is mostly related to the survival and virulence of mycobacteria, including Rv3041c (NDB), FecB (SBP), and IrtA/IrtB in Mtb [24,53]. Previous studies have shown that Rv1085c, as a homologous protein for MSMEG5257 in Mtb, is required for Mtb heme utilization [54]. The expression of ABC transporter proteins in Ms_Δ*msmeg5257* was downregulated, and this is potentially attributed to alterations in iron metabolism. MSMEG3636, defined as a ferric iron-binding periplasmic protein of the ABC transporter, was downregulated in Ms_Δ*msmeg5257*. The metal element iron is essential for life activities, playing a crucial role in protein and nucleic acid synthesis, respiration, and other physiological processes [55,56]. Our RNAseq study showed a significant impact on the expression of proteins related to the biosynthesis of siderophore group nonribosomal peptides upon the deletion of *msmeg5257* in 7H9 media. The 7H9 media contains about 130 μM Fe^3+^, which is characteristic of an iron-rich environment [57]. The statement suggests a potential correlation between *msmeg5257* and the uptake of iron ions. The expression of *msmeg5257* may be upregulated in environments with low iron concentrations. We recognize that we did not examine the mRNA levels of *msmeg5257* in iron-poor media, but based on the phenotypic experiment’s precedence, we opted to characterize *msmeg5257* via the growth assay of the mutant strain in iron-poor environments. Corresponding to this, we observed that the capacity to uptake iron ions is diminished in environments with low iron levels for the Δ*msmeg5257* strain, which hampers its growth in the low-iron medium (1 μM Fe^3+^). The implication is that MSMEG5257 may exhibit functionality in an iron-depleted environment. Mycobacteria are classified as intracellular bacteria. Although determining the concentration of iron ions within intracellular organelles is challenging, the previous study has demonstrated the presence of approximately 600 nM hemin in IMR90 human lung fibroblasts and 400 nM hemin in HEK293 cells [58]. The implication is that *msmeg5257* may play a role during a specific stage of bacterial infection within the host. Furthermore, it may help maintain the integrity of the Ms cell envelope and contribute to modulating the host response during infection. However, these speculations necessitate further empirical evidence. Investigating the physiological and immune functions of MSMEG5257 is imperative and holds great significance in unraveling the intricate roles played by mycobacterial membrane proteins. It is suggested that the culture of Mtb with controlled iron ion content is very important for the elucidation of drug resistance, target analysis, and virulence studies in vitro.

In mycobacteria, transmembrane proteins, which belong to the α-helical and β-barrel structural classes, play crucial roles in material transportation and energy metabolism [17]. For instance, with respect to CpnT, a channel protein located in the outer membrane of Mtb [59], the N-terminal domain forms an embedded channel that facilitates efficient glycerol uptake [60]. In our study, we established that MSMEG5257 is located in the cytomembrane of Ms, and we have observed that MSMEG5257 has a free N-α-helix in the cytoplasm. The implication is that *msmeg5257* potentially binds and transports small molecules or ions to ABC transporters via the free N-α-helix. For instance, the Rv1085c homologue is reportedly involved in Mtb’s ESX-4 secretion system [61,62]. This finding provides further evidence that *msmeg5257* exerts its regulatory function via the modulation of ABC transporters. Furthermore, a free α-helix in the MSMEG5257 system offers valuable insights for designing an inhibitor targeting this function, such as mycobacterial membrane protein large 3 (MmpL3) [63].

The physiological and immune mechanism of *msmeg5257* needs to be further investigated to gain a deeper understanding of the intricate membrane system of mycobacteria. Considering the homology between Ms and Mtb, do genes with analogous functions also exist in Mtb? For example, does the *msmeg5257* homolog *Rv1085c* exhibit a similar characteristic? Although a previous study has reported the involvement of *Rv1085c* in the ESX-4 secretion system [54], the precise immune function of *Rv1085c* during Mtb infection remains elusive and warrants further investigation. Identifying such genes can provide valuable therapeutic targets, thereby expanding possibilities for clinical drug combinations.

## 5. Conclusions

This study suggests that the deletion of *msmeg5257* increased the growth rate of Ms by decreasing the expression of ABC transport proteins. Our data provide insights that *msmeg5257* plays a role in maintaining Ms metabolic homeostasis, such as the bacterial uptake of iron ions. To elucidate the role of membrane proteins from the mycobacterial complex in physiological and immune processes, it is imperative to investigate the *msmeg5257* function in drug resistance and immune mechanisms. This will benefit the development of targeted drugs or agents for mycobacterium with respect to T cell immunity processes. These findings will facilitate the development of treatment plans for Mtb.

## Data Availability

The data are available in the article or its Supplementary Materials.

## Supplementary Materials

The following supporting information can be downloaded at https://www.mdpi.com/article/10.3390/microorganisms12040770/s1, Figure S1: Construction of *msmeg5257* deletion strain.; Figure S2: The growth curve of wild-type Ms strains; Figure S3: Revisions in the mRNA expression levels of genes implicated in iron transport within the *msmeg5257* mutant strains. Table S1: List of primers, strains, plasmids, antibodies, and ELISA kits used in the study; Table S2: Drug name and concentration of drug susceptibility test; Table S3: Drug susceptibility test of *msmeg5257* mutant strains; Table S4: List of differentially expressed genes in *msmeg5257* deletion strain compared with the wild-type strain; Table S5: List of differentially expressed genes in *msmeg5257* complementation strain compared with the vector wild-type strain; Table S6: List of differentially expressed proteins in *msmeg5257* mutant strains.
