# Supplementary material for: Impact of MSMEG5257 Deletion on Mycolicibacterium smegmatis Growth
Source: Microorganisms. 2024 Apr 11;12(4):770. doi: 10.3390/microorganisms12040770 (PMC11052289; doi:10.3390/microorganisms12040770)
Supplement: Supplementary file 1 [file microorganisms-12-00770-s001.zip › microorganisms-2882301-supplementary.pdf]

Supplementary materials

Table S1. List of primers, strains, plasmids, antibodies, and ELISA kits used in the study.

List of primers used in the study for cloning purposes		
**Name**	**Primers (5′-3′)**	**Usage**
OE-*msmeg5257*-Fwd	TGATTAACTTTATAAGGAGGAAAAAC ATATGATGACCGCGCCGATCGACG	Construction of the overexpression of *msmeg5257* in Ms
OE-*msmeg5257*-Rev	TCTTCAGAAATAAGTTTTTGTTCCAAA GCTTGGCGTAGTCCGGC	
sgRNA-*msmeg5257*-Fwd	GGGACCCAGTTCTTCGGAAAACCA	Construction of the deletion of *msmeg5257* in Ms
sgRNA-*msmeg5257*-Rev	AAACTGGTTTTCCGAAGAACTGGG	
Cas9-Asp-Fwd	AGCATCGGCCTGGACATCGGCACGAA CTCGGTGGGCT	
Cas9-Asp-Rev	GTTCGTGCCGATGTCCAGGCCGATGCT GTACTTCTTGTCCATATGTATAT	
Cas9-His-Fwd	TACGACGTGGACCACATCGTGCCGCA GTCGTTCCTGAAG	
Cas9-His-Rev	CTGCGGCACGATGTGGTCCACGTCGTA GTCGCTCAGCCG	
**List of primers used in the study for RT-PCR**		
msmeg4509-Fwd	GTCGATCCTTCCCGGCAACC	
msmeg4509-Rev	GGGTCGGAGTACAGCGTGTTC	
msmeg4510-Fwd	AGTACGTCGTGGCGATGCTC	
msmeg4510-Rev	GGTGAAGACGATGTAGGCGCC	
msmeg4511-Fwd	CGGTCTGCCCAAAGGTGTTC	
msmeg4511-Rev	CGGTCAGGTAGCCGATGTC	
msmeg4512-Fwd	GGTGTGGTGCTCGTCGGAT	
msmeg4512-Rev	ACCTGGTGCCATTGCTCGG	
msmeg4513-Fwd	ACTGCGATCTGGCGGTGAC	
msmeg4513-Rev	GGTGCGTCCGTCCTGGTTGA	
msmeg4515-Fwd	AGGCACCACTGGCTGGATC	

msmeg4515-Rev	TCGTCGTATCGGTCAGGTCG
msmeg4516-Fwd	CCGCCGACGACGTCTATCT
msmeg4516-Rev	CCAAGGGTTCCCACGCACAC
msmeg1373-Fwd	CAAGGTGATGGCGGCGATGG
msmeg1373-Rev	AGGCGCTGAAGTCGGTGAG
msmeg1613-Fwd	GCATGAAGCCCGCGAAAGTC
msmeg1613-Rev	TCGTGGTCAGCGCCAGGTA
msmeg1709-Fwd	ATCTGAGCGTGGGTTCGGTG
msmeg1709-Rev	CCGGGTCGTTGATCGGTATCG
msmeg2635-Fwd	CCTACCTCGCCCACAACACC
msmeg2635-Rev	GGGAAGAACGCCATGAGCACG
msmeg2846-Fwd	GCCCAGGTGCTGCTCAAGAA
msmeg2846-Rev	GACCGGGAGAACACGGTCTC
msmeg3999-Fwd	ACCGAGTCTGATGCCCGCAA
msmeg3999-Rev	TCGTCCACGAGCAACCCCT
msmeg5102-Fwd	CGCCCTGAGGTCCTGATGC
msmeg5102-Rev	GTACGCCGTGAAGTTGCCG
msmeg5368-Fwd	AGATCAACGTCGGCTTCGCC
msmeg5368-Rev	CGGATTGCTGAACGCTGCCT
msmeg5370-Fwd	GTGGATCTCCTGGCCGTCC
msmeg5370-Rev	ACTGCCCTTGGGGAATCGC
msmeg5660-Fwd	CCGCAAGGACCTCTACGCAC

msmeg5660-Rev	ACCAGCACCACGACACCCA	
msmeg6332-Fwd	GCGGCGAGCATCATCTTCAC	
msmeg6332-Rev	GCGGCAGTTCGACTTTGAGC	
msmeg6333-Fwd	CAAACTCGCCGACCGCTATC	
msmeg6333-Rev	GCCTCGTCGATGTCATGGG	
msmeg6334-Fwd	CGCCGAGGACAAGGACACC	
msmeg6334-Rev	CGCTGATCGCGACGAAGTTG	
16S-Fwd	GGGCGATACGGGCAGACTA	
16S-Rev	CACGGATCCCAAGGAAGGA	
**List of strains and plasmids used in the study**		
**Strains and plasmids**	**Description**	**Reference**
E. coli DH5α	*E. coli* DH5α for molecular cloning	CWBIO
M. smegmatis mc^{2}155	*M. smegmatis* mc^{2}155 parental strain	ATCC
Ms_*vec*	The empty vector in Ms	This study
Ms_Δ*msmeg5257*	Deletion of *msmeg5257* in Ms	This study
Ms_OE-*msmeg5257*	Overexpression of *msmeg5257* in Ms	This study
Ms_C-Δ*msmeg5257*	Complement of *msmeg5257* in Ms_Δ*msmeg5257*	This study
**Plasmids**	**Description**	**Reference**
pSUM-Kan-MCS2	Cloning vector	109379, ADDGENE
pSUM-OE-*msmeg5257*	*msmeg5257* Overexpression plasmid	This study
pRH2521	Cloning vector	84380, ADDGENE
pRH2502	dCas9-induced expression plasmid	84379, ADDGENE
pRH2502-Cas9	Cas9-induced expression plasmid	This study
pRH2521-sgRNA-*msmeg5257*	sgRNA-induced expression plasmid	This study
**List of antibodies used in the study**		
**Name**	**Identifier**	**Reference**
Anti-MSMEG5257-antibody	NWTSVTARKWMKR	This study

Anti-rabbit IgG (H+L)	14708	CTS
Goat Anti-rabbit IgG (H&L)	Y1055	LABLEAD
Goat Anti-mouse IgG H&L (HRP)	ab205719	ABCAM

Table S2 Drug name and concentration of drug susceptibility test.

Drugs	Concentrations (μg/mL)
Bedaquiline (BDQ)	0.008, 0.015, 0.03, 0.06, 0.12, 0.25, 0.5, and 1.
Ethambutol (EMB)	0.25, 0.5, 1, 2, 4, 8, 16, and 32.
Rifampicin (RIF)	0.03, 0.06, 0.12, 0.25, 0.5, 1, 2, 4, and 8.
Amikacin (AMI)	0.25, 0.5, 1, 2, 4, 8, and 16.
Levofloxacin (LEVO)	0.12, 0.25, 0.5, 1, 2, 4, and 8.
Moxifloxacin (MXF)	0.06, 0.12, 0.25, 0.5, 1, 2, and 4.
Delamanid (DLM)	0.008, 0.015, 0.03, 0.06, 0.12, 0.25, and 0.5.
Linezolid (LZD)	0.06, 0.12, 0.25, 0.5, 1, and 2.
Clofazimine (CFZ)	0.03, 0.06, 0.12, 0.25, 0.5, 1, and 2.
Ethionamide (ETO)	0.5, 1, 2, 4, and 8.
Rifabutin (RFB)	0.06, 0.12, 0.25, 0.5, 1, and 2.
Isoniazid (INH)	0.025, 0.05, 0.1, 0.2, 0.4, 0.8, 1.6, 3.2, 6.4, and 12.8.

Table S3 Drug susceptibility test of *msmeg5257* mutant strains.

	Minimum Inhibitory Concentration (μg/mL)	
Drugs	Ms_WT	Ms_Δ*msmeg5257*
Bedaquiline (BDQ)	0.03	0.015
Clofazimine (CFZ)	0.5-1	0.25-0.5

Supplementary Figure

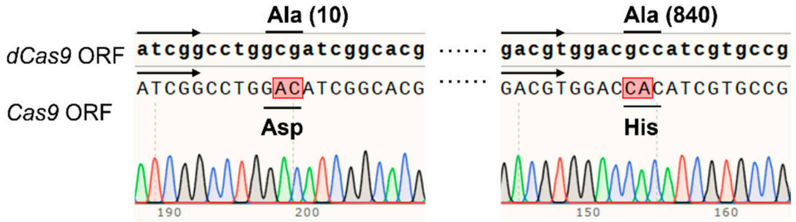

(a)

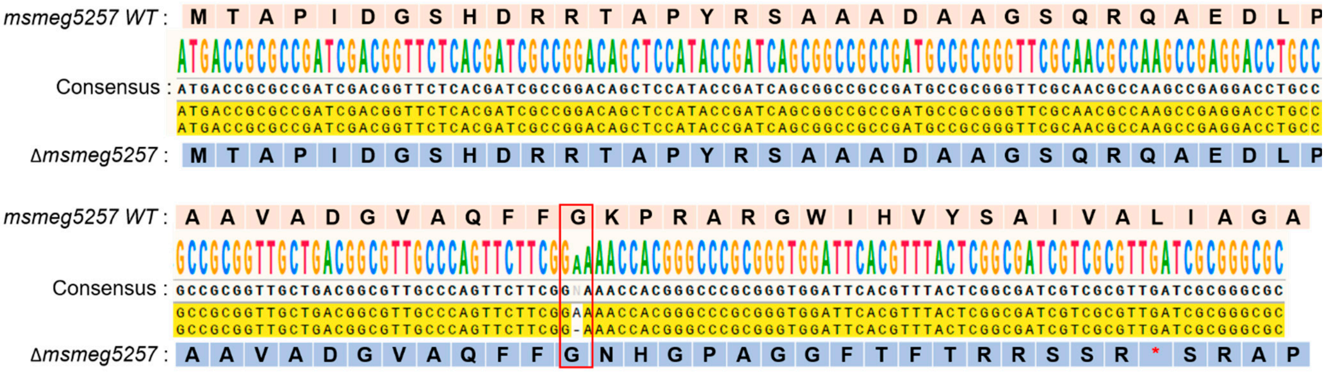

(b)

Supplementary Figure S1. Construction of *msmeg5257* deletion strain. (**a**) The sequencing outcome of the pRH2502-Cas9 plasmid**.** The Ala at position 10 changed to Asp, and Ala at position 840 changed to His in the *dCas9* amino acid (AA) sequence of pRH2502-dCas9. ORF, open reading frame. (**b**) The alignment results of the whole-genome sequencing for Ms_Δ*msmeg5257*. Yellow: identical base; -: location of the base deletion.

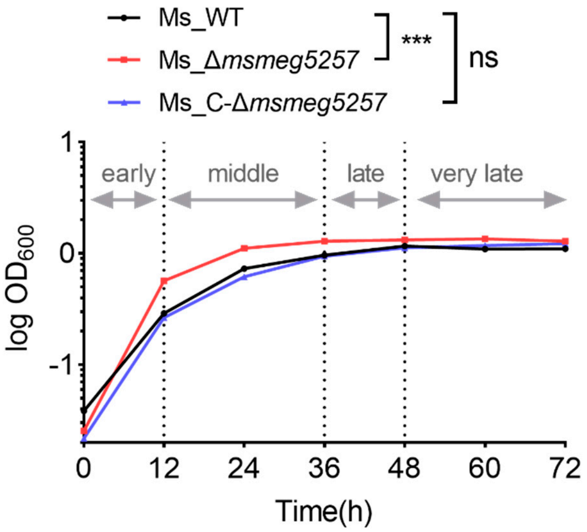

Supplementary Figure S2. The growth curve of wild-type Ms strains. The growth curve of Ms was divided into early (0-12 h), middle (13-36 h), late (37-48 h), and very late (> 48 h) stages. ***: p values < 0.001 as a very significant difference; ns: no difference; WT: Ms_WT; Δ: Ms_Δ*msmeg5257*; C-Δ: Ms_C-Δ*msmeg5257*.

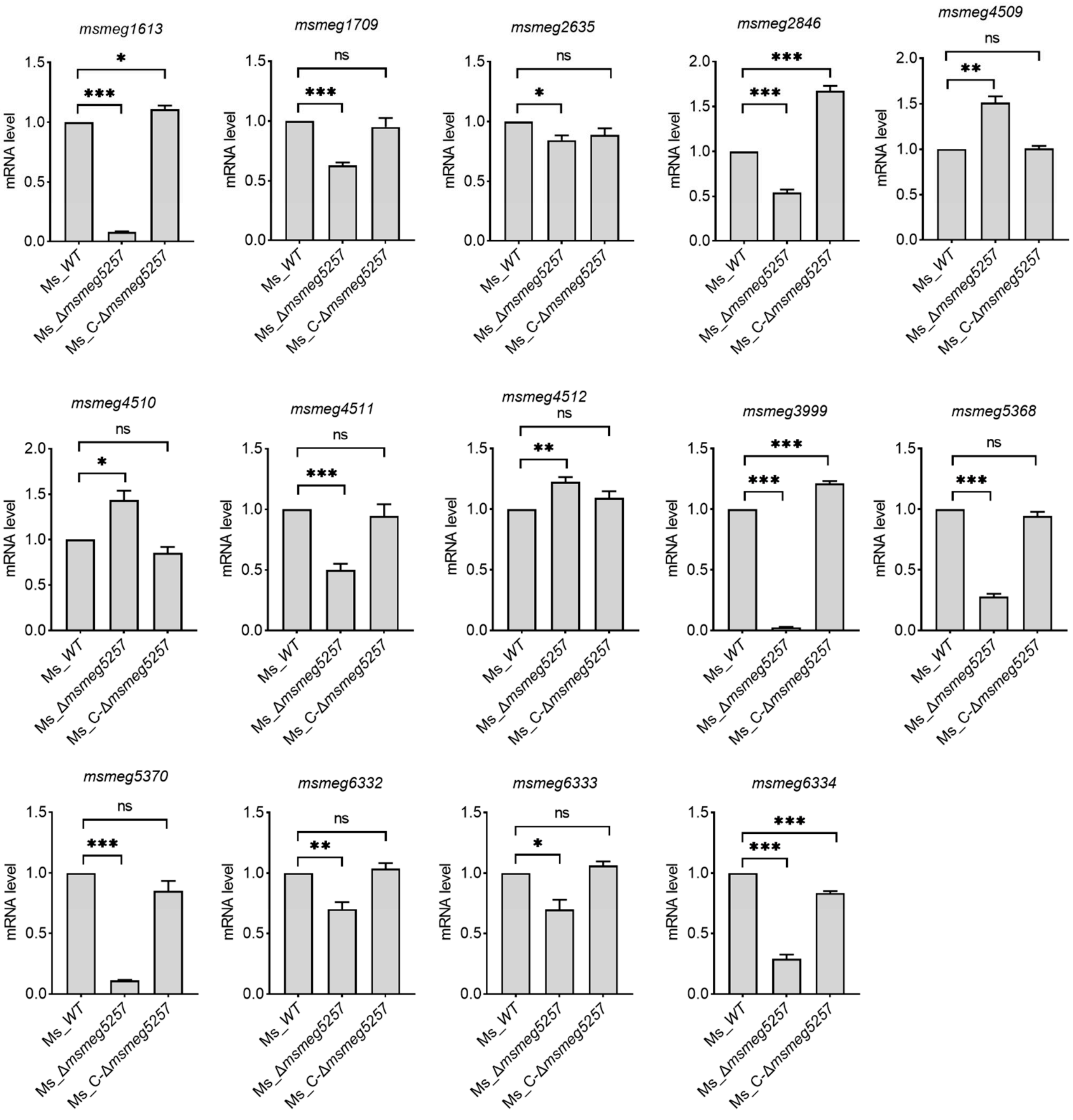

Supplementary Figure S3. Revisions in the mRNA expression levels of genes implicated in iron transport within the *msmeg5257* mutant strains. *: *p* values < 0.05; **: *p* values < 0.01; ***: *p* values < 0.001; ns: no difference.
